# The Regulating Effect of Autophagy-Related MiRNAs in Kidney, Bladder, and Prostate Cancer

**DOI:** 10.1155/2021/5510318

**Published:** 2021-04-22

**Authors:** Kai Huang, Xiaoxin Sun, Haotian Wu, Jun Zhao, Yuli Jian, Zhongyang Xu, Shujing Wang, Deyong Yang

**Affiliations:** ^1^Department of Urology, First Affifiliated Hospital of Dalian Medical University, Dalian, China; ^2^College of Integrative Medicine, Dalian Medical University, Dalian, China; ^3^Department of Biochemistry, Institute of Glycobiology, Dalian Medical University, Dalian, China

## Abstract

Autophagy is a treatment target for many disorders, including cancer, and its specific role is becoming increasingly well known. In tumors, researchers pay attention to microribonucleic acids (miRNAs) with regulatory effects to develop more effective therapeutic drugs for autophagy and find new therapeutic targets. Various studies have shown that autophagy-related miRNAs play an irreplaceable role in different tumors, such as miR-495, miR-30, and miR-101. These miRNAs are associated with autophagy resistance in gastric cancer, non-small cell lung cancer, and cervical cancer. In recent years, autophagy-related miRNAs have also been reported to play a role in autophagy in urinary system tumors. This article reviews the regulatory effects of autophagy-related miRNAs in kidney, bladder, and prostate cancer and provides new ideas for targeted therapy of the three major tumors of the urinary system.

## 1. Introduction

Microribonucleic acids (miRNAs) are a kind of small noncoding RNA with a length of 17–25 nucleotides that regulate the expression of many genes by base-pairing the complementary sequences of the 3′-untranslated region (3′-UTR) [1, 2]. Disorders of gene expression are the main signs of cancer, and miRNAs play an important role in regulating gene expression programs, which are the basis of pathological cell processes (including cancer) [3, 4]. Many human miRNA genes are located at fragile sites that are subject to translocation, amplification, deletion, or mutation in cancer [5]. These molecules usually reduce the mRNA's translation and stability, including those genes that mediate tumorigeneses, such as apoptosis, cell cycle regulation, stress response, differentiation, and invasion [6–8]. On the one hand, the reduction or suppression of miRNA, which is a tumor suppressor gene, leads to the formation of tumors. For example, in renal cell carcinoma (RCC), in vivo and in vitro experiments have shown that inhibition of miR-363 will promote the progression of RCC [9]. Similarly, miR‐487a‐3p functions as a new tumor suppressor in prostate cancer by targeting CCND1 [10]. On the other hand, the amplification or overexpression of a miRNA that has an oncogenic role could also contribute to the formation of tumors. Chang et al. demonstrated that a more invasive bladder cancer (BC) phenotype was significantly and positively correlated with miR-516a overexpression in BC patients [11].

**Figure 1:**
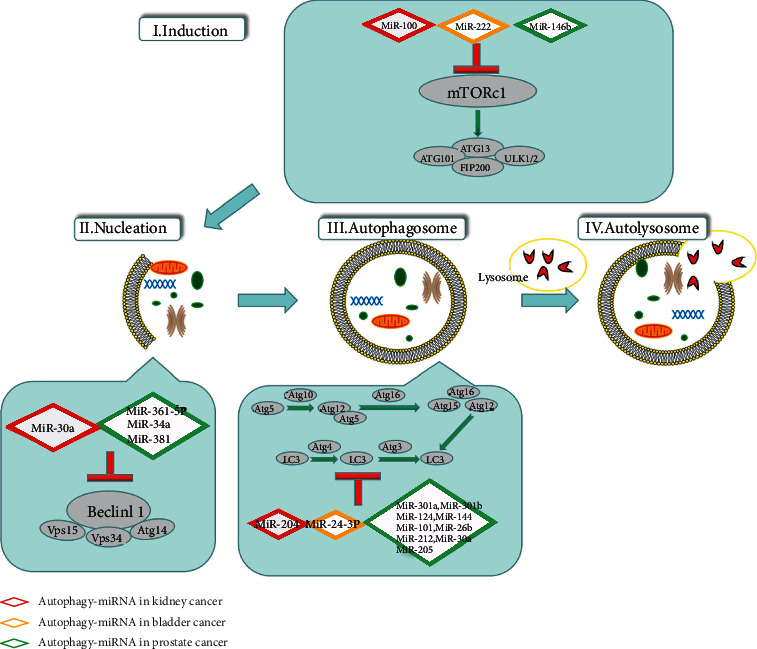
The regulation of autophagy-related miRNAs in kidney cancer, bladder cancer, and prostate cancer. The red diamond box represents kidney cancer, the orange diamond box represents bladder cancer, and the green diamond box represents prostate cancer. MiRNAs play a regulatory role by influencing autophagy pathway-related proteins (such as LC3).

While defects in the process of autophagy may prompt healthy cells to acquire malignant characteristics, the autophagy response may be an indispensable condition for the survival, proliferation, and growth of cancer cells in the microenvironment [12]. A large number of studies have recently reported the initiation of miRNA-regulated autophagy in tumors [13]. In the process of tumorigenesis and development, miRNAs induce angiogenesis and participate in tumor cell metabolism and other biological, behavioral changes through the regulation of tumor invasion and autophagy-related metastases [14, 15]. With the continuous progress of epigenetic research, the roles of autophagy-related miRNAs in colorectal cancer, lung cancer, osteosarcoma, gastric cancer, and brain tumors are becoming more and more well-known [16–20]. However, the specific mechanisms of autophagy-related miRNAs in kidney cancer, bladder cancer, and prostate cancer are still unclear. This study has summarized the role of autophagy-related miRNAs in kidney, bladder, and prostate cancer (Figure 1), which provides a sufficient theoretical basis for better-targeted regulation and therapy future.

## 2. MiRNA in Autophagy Pathway

There are three types of autophagy in morphology and mechanism: macroautophagy, microautophagy, and chaperone-mediated autophagy [21]. Macroautophagy (hereinafter referred to as autophagy) refers to the formation of autophagosomes in which damaged mitochondria and nuclear fragments are encapsulated by a double-layer membrane in the cytoplasm and then fuses with lysosomes to form autophagolysosomes and degrade their functional content [22]. It should be noted that the essential role of autophagy is the turnover of proteins and organelles, which have a variety of physiological and pathological functions [23].

### 2.1. Autophagy-Related MiRNAs and Nonneoplastic Diseases

Autophagy has become the focus of neurodegenerative disease research under normal physiological conditions due to its functions [24]. For example, cellular MTORC1 activity is vital for longevity. Reducing TOR signaling can improve health by improving antistress and carefully regulating metabolism [25]. It also participates in regulating the metabolism of muscle energy, essential for the human body's everyday life [26]. Autophagy, therefore, has a specific position in the maintenance of daily life activities [27]. Besides, while focusing on autophagy, miRNA, as the upstream of regulation, also plays a role in neurodegeneration and coronary heart disease [28, 29].

### 2.2. Autophagy-Related MiRNAs and Tumors

Autophagy, as a survival pathway and quality control mechanism, participates in normal cell physiological metabolism, provides biological materials and energy to cope with stress, also contributes to tumorigenesis and tumor development by removing damaged proteins and organelles, and prevents tumorigenesis [30–32]. Once the tumor progresses to a later stage, the pressure from the tumor environment will follow. Limited angiogenesis, insufficient nutrition, and hypoxia can affect tumor cells to some degree. Autophagy, dynamic degradation, and recycling systems will contribute to tumor development [33]. It is, therefore, necessary to analyze indepth the specific mechanism of autophagy. Recent studies have shown that noncoding RNA regulates autophagy by affecting the expression of related genes [34]. A class of miRNAs that first appeared in many noncoding RNA studies continues to explore their regulatory pathways in autophagy [29, 35]. It focuses on miRNA involved in autophagy induction, nucleation, and prolongation [36–38]. However, the mechanism of autophagy-lysosome formation and cleavage is relatively less.

In conclusion, as miRNA's regulatory role in the autophagy process continues to be understood, these studies may play an irreplaceable role in understanding tumor initiation, biological behavior, treatment, and drug resistance during treatment. This study reviews the relevant regulatory effects of autophagy-related miRNAs (Table 1) in the three major urinary tract tumors, including kidney cancer, bladder cancer, and prostate cancer, which will help us develop promising cancer biomarkers and therapeutic targets.

## 3. The Role of Autophagy-Related MiRNAs in Kidney Cancer

Although the diagnosis and treatment of renal cell carcinoma (RCC) have improved over the last two decades, RCC is still a fatal malignant tumor in the urinary system [61]. Clear cell renal cell carcinoma (ccRCC) accounts for the most significant proportion, and papillary and mixed renal eosinophilic tumors are increasingly common in clinics [62]. RCC incidence and mortality vary significantly around the world, and the current demand for accurate biomarkers has not been met [63].

As mentioned above, autophagic-related miRNAs may affect tumor pathogenesis. Previous studies in RCC found that VHL-regulated miR-204 inhibits tumor growth by inhibiting LC3-mediated autophagy in ccRCC [39]. VHL is the most common tumor suppressor factor in ccRCC by miR-204 regulation that affects the autophagy extension phase after nucleation to inhibit further autophagy [64]. Hall et al. found that TRPM3 and miR-204 established a regulatory loop to control the oncogenic autophagy of ccRCC and TRPM3. Ca2+ and Zn2+ inhibited direct target miR-214 inhibition of LC3 [65]. MiR-214 acts as an inhibitor of autophagy. Conversely, autophagy can also suppress tumor-related phenotypes due to the dual nature of autophagy. For example, upregulating miR-100 can increase autophagy and inhibit the migration and invasion of RCC cells by targeting NOX4 and inactivating the MTOR pathway [40].

Sorafenib is a well-known antitumor drug in RCC, and chemotherapy-induced autophagy activation usually helps cancer resistance [66, 67]. Sorafenib is effective for early-stage tumors, but unfortunately, the recurrence of RCC due to sorafenib resistance is not unusual. For example, the long noncoding RNA KIF9-AS1 regulates the transformation of growth factor-b and autophagy signals through miR-497-5p to enhance RCC chemotherapy resistance. The expression of miR-497-5p decreased, and the expression of ATG9A in the corresponding autophagy pathway elevated. RCC is resistant to sorafenib [43]. MiR-30a also mediates autophagy inhibition to make RCC cells sensitive to sorafenib, and miR-30a is an effective autophagy inhibitor by downregulating BECN1 [41]. The same hsa-miR-335 also plays a role in inhibiting autophagy. In the study of Yan et al., hsa-circ_0035483 can enhance gemcitabine resistance by activating autophagy in TK10 and UO31 cells. The expression of hsa-circ_0035483 was negatively correlated with the expression of hsa-miR-335. After knocking out hsa-circ_0035483, the expression of hsa-miR-335 increased, CCNB1 expression decreased, and gemcitabine resistance decreased [42].

From the current research, the main focus is on the direct or indirect effects of autophagy on RCC, in which miRNAs play an indispensable role, such as miR-24, miR-214, and miR-497-5p. Therefore, affecting the role of miRNA as a regulatory molecule may become an effective treatment strategy for RCC. However, due to the dual nature of autophagy, the security of this strategy still requires us to be cautious.

## 4. The Role of Autophagy-Related MiRNAs in Bladder Cancer

Bladder cancer (BC) is the eleventh most common cancer globally, and 500,000 people are diagnosed with bladder cancer every year [68, 69]. Three-quarters of the cases were nonmuscular invasive bladder cancer, and the rest were muscular invasive bladder cancer (MIBC) or metastatic bladder cancer [70]. Although BC patients' incidence and mortality rates are still high and the systemic treatment of BC has not changed for more than 30 years, people are still developing predictive biomarkers and appropriate combination programs to improve the use of therapy [71, 72].

As Patel et al. said, new approaches to the treatment of BC continue to be urgently needed, given the still limited options available to patients at an early stage of the disease [72]. Here, we summarize the related reports; for example, miR-24-3p is highly expressed in BC tissues, and DEDD is lowly expressed in BC tissues. MiR-24-3p promotes cell proliferation, migration, and invasion, inhibits cell apoptosis, and participates in autophagy of BC cells through LC3, DEDD, and p62 [46]. MiR-154 acts as a tumor suppressor in BC by targeting ATG7, a critical molecule in the autophagy process. The expression of ATG7 is negatively correlated with the expression of miR-154 in BC tissue [73]. It is speculated that miR-154 can also affect the autophagy pathway, and further research is needed in the future. Similarly, in the Xiaoping Liu et al. study, we learned that the downregulation of miR-221 induces autophagy and inhibits the migration and invasion of BC cells TP53INP1/p-ERK axis [44]. Given the current outlook for BC treatment, there is an urgent need to study new targets.

Autophagy-related miRNAs as a target may have a bright future in the treatment of BC. However, unfortunately, few studies on the mechanism of autophagy-related miRNAs in BC and further exploration are still needed.

## 5. The Role of Autophagy-Related MiRNAs in Prostate Cancer

Prostate cancer (PCa) is the most common malignant tumor in men, and more than 1.2 million men worldwide were diagnosed with PCa in 2018 [74, 75]. Most patients with PCa generally undergo localized radical prostatectomy, radiation, and chemotherapy after the diagnosis [76–78]. Among them, tumor drug resistance has become one of the crucial reasons for the poor treatment effect, so an indepth study of drug resistance-related mechanisms is essential [79].

MiRNAs are small regulatory molecules that can also be used as contributors to cancer cells' resistance to commonly used anticancer drugs [80, 81]. Here, we will explore the role of autophagy-related miRNAs in PCa, including the relationship between miRNA in the autophagy pathway and tumor progression and drug resistance. The regulation of autophagy is complex and flexible. It is first reflected in the related autophagy induced under hypoxic conditions. Yi Ma et al. indicate that the regulation of miR-96 is in dynamic equilibrium in hypoxia, and inhibition of MTOR by up-regulating miR-96 may promote autophagy. However, ectopic overexpression of miR-96 above a certain threshold may disrupt the balance and inhibit autophagy, so the biphasic regulation of autophagy by miR-96 affects PCa cell proliferation and tumor growth [54]. MiR-124 and miR-144 are two hypoxia-responsive miRNAs, which can reduce hypoxia-induced autophagy and enhance PCa cells' radiosensitivity by reducing PIM1 [52]. Similarly, miR-30a and miR-205 are also two hypoxia-responsive miRNAs, simultaneously targeting TP53INP1 and inhibiting its expression. The miR-30a/miR-205/TP53INP1 axis is involved in regulating autophagy and radiosensitivity [59]. Next, the complexity of miRNA regulation is not only reflected in hypoxia-related miRNAs. MiR-34a, as a tumor suppressor miRNA, induces autophagy in PCa cells that is antiproliferative, a combination of autophagy and apoptosis. The role is the cause of miR-34a-mediated inhibition of prostate tumor growth [47], while miR-212 negatively regulates starvation-induced PCa cell autophagy by targeting sirtuin1 (SIRT1) [57]. Similarly, miR-101 is expressed low in PCa. The autophagy inhibition of miR-101 mimics was found to enhance the cytotoxic effect of *Tripterygium wilfordii* on PCa cells [53]. John Clotaire and others also explored the role of miRNA in the autophagy pathway and found that miR-26b mainly inhibits PCa cells' autophagy by targeting ULK2 [56]. And, recent research reports that miR-381 promotes autophagy and apoptosis of PCa cells by inhibiting the RELN-mediated PI3K/AKT/mTOR signaling pathway, which shows that autophagy-related miRNAs has excellent potential for PCa treatment [58]. The prospect of the application provided a sound theoretical basis for the promotion of the clinical application.

Here, we summarize the latest advances in the role of autophagy-related miRNAs in regulating PCa, such as the regulation of miRNAs on tumor autophagy resistance. Therefore, in future research, miRNA may be used as a good monitoring indicator in the field of tumor autophagy resistance. In summary, the investigation of autophagy-related miRNAs molecular mechanisms in the regulation of PCa tumorigenesis or progression may provide novel therapies of PCa.

## 6. Prospects for Targeted Therapy of Autophagy-Related MiRNAs in Kidney, Bladder, and Prostate Cancer

Molecular targeted therapy can prevent cancer growth, development, and metastases by affecting specific molecules. It is an effective strategy for treating cancer by molecular therapy alone or standard chemotherapy drugs [82]. Due to their small size, small molecule inhibitors can potentially bind to a wider range of extracellular and intracellular targets so that they will have great potential in tumor treatment in the next 10 years [83]. As one of many tumor pathways, the autophagy pathway is targeted at more and more molecules on the autophagy pathway [84]. Interfering with autophagy represents a reasonable treatment strategy [85, 86]. So, Levy et al. summarized the design and results of previous clinical trials, the development of autophagy-dependence and response biomarkers, and the role of autophagy in chemotherapy resistance and discussed how to use autophagy to maximize the treatment response of cancer [87].

At present, in the diagnosis and treatment of kidney, bladder, and prostate cancer, both the classical autophagy inhibitors, such as chloroquine and hydroxychloroquine, and the preclinical development of more autophagy-related experimental compounds are determined by the duality of autophagy. It is necessary for us to determine the factors that cause the condition-dependent behavior of autophagy, study different genes and signal pathways, and identify molecular markers to understand how autophagy works at the molecular level, so as to be more helpful to tumor targets towards treatment [88, 89]. As mentioned above, the key role of miRNA in tumors is undoubted. Small molecule inhibitors targeting specific microribonucleic acids (SMIRs) will become a new way of treating tumors [45, 90]. Therefore, how to regulate the miRNA in the autophagy pathway to achieve the purpose of treating tumors will become a major difficulty.

In conclusion, at this stage, there is still a long way to go before miRNA-based drugs can be used to treat kidney, bladder, and prostate cancers at this stage. Encouragingly, to study the regulation of a large number of autophagy-related miRNAs in primary urology cancers, the control effect is constantly being studied and elucidated, laying the foundation for subsequent autophagy-related miRNAtargeted therapies. However, the specific molecular mechanisms, drug development, and clinical trials to verify the safety and effectiveness of drugs still require a lot of efforts to study and explore.

## Figures and Tables

**Table 1:** Autophagy-related miRNAs in Urological tumors.

Tumor	MiRNA	Expression（up/down）	Effect	Target Gene	Ref
kidney cancer					
	MiR-204	Down	Promote autophagy	LC3	[39]
	MiR-100	Down	Inhibit autophagy	MTOR	[40]
	MiR-30a	Down	Promote autophagy	BECN1	[41]
	Hsa-miR-335	Down	Promote autophagy		[42]
	MiR-497-5p	Down	Promote autophagy	ATG9A	[43]

Bladder cancer					
	MiR-221	Up	Inhibit autophagy		[44]
	MiR-222	Up	Inhibit autophagy	MTOR	[45]
	MiR-24-3p	Up	Promote autophagy	LC3	[46]

Prostate cancer					
	MiR-34a	Down	Inhibit autophagy		[47]
	MiR-361-5p	Up	Inhibit autophagy	LC3, BECN1	[48]
	MiR-205	Up	Inhibit autophagy	TP53INP1	[49]
	MiR-146b	Up	Inhibit autophagy	MTOR	[50]
	MiR-143	Up	Inhibit autophagy	ATG2B	[51]
	MiR-124, MiR-144	Up	Inhibit autophagy	PIM1, LC3	[52]
	MiR-101	Up	Inhibit autophagy	LC3	[53]
	MiR-96, MiR-34a	Up	Inhibit autophagy	ATG7	[54]
	MiR-34a	Down	Promote autophagy	ATG4B, BECN1, LC3	[55]
	MiR-26b	Up	Inhibit autophagy	ULK2, LC3	[56]
	MiR-212	Down	Promote autophagy	SIRT1, LC3	[57]
	MiR-381	Up	Promote autophagy	RELN, LC3, BECN1	[58]
	MiR-30a, MiR-205	Down	Inhibit autophagy	LC3	[59]
	MiR-301a,	Up	Promote autophagy	NDRG2	[60]
	MiR-301b				

## Data Availability

The data used to support the findings of this study are available from the corresponding author upon request.
